# Supplementary material for: Phenolic Profiling and In-Vitro Bioactivities of Corn (Zea mays L.) Tassel Extracts by Combining Enzyme-Assisted Extraction
Source: Foods. 2022 Jul 20;11(14):2145. doi: 10.3390/foods11142145 (PMC9320485; doi:10.3390/foods11142145)
Supplement: Supplementary file 1 [file foods-11-02145-s001.zip › foods-1792186-supplementary.pdf]

Table S1. Central composite design and response for TPC value in corn tassel extract by cellulase-assisted method

Run	Incubation time (h, χ_1)	Cellulase concentration (U/g, χ_2)	TPC (mg/g)
1	36	1500	9.49
2	12	1000	6.93
3	12	1000	7.01
4	60	500	7.79
5	60	2500	8.94
6	48	500	8.25
7	12	2500	6.87
8	36	2500	9.66
9	12	500	5.98
10	36	500	8.82
11	36	1000	9.29
12	36	500	8.93
13	36	1000	9.21
14	36	1500	9.58
15	12	500	5.96
16	60	500	7.80
17	60	2500	8.90
18	12	2500	7.87
19	36	1500	9.48
20	12	500	5.67
21	36	1500	9.50
22	60	1500	8.81
23	12	2500	7.87
24	24	1500	8.89
25	48	1000	9.02
26	36	1500	9.49
27	36	1000	9.24
28	12	2500	7.88
29	48	2500	9.29
30	24	2500	8.28

Table S2. Central composite design and response for TPC value in corn tassel extract by protease-assisted method

Run	Incubation time (h, χ_1)	Protease concentration (U/g, χ_2)	TPC (mg/g)
1	36.00	1500.00	7.93
2	12.00	500.00	5.82
3	60.00	500.00	7.81
4	24.00	500.00	6.65
5	60.00	2500.00	8.30
6	48.00	500.00	8.40
7	12.00	2500.00	6.42
8	36.00	2500.00	7.83
9	12.00	500.00	4.75
10	36.00	500.00	8.69
11	36.00	500.00	8.15
12	36.00	1000.00	8.20
13	36.00	500.00	8.52
14	36.00	1500.00	7.80
15	12.00	500.00	4.67
16	60.00	500.00	7.78
17	24.00	2500.00	6.91
18	12.00	2500.00	5.19
19	36.00	1500.00	7.94
20	12.00	500.00	5.17
21	36.00	1500.00	7.92
22	60.00	1500.00	7.71
23	12.00	2500.00	5.87
24	24.00	1500.00	6.79
25	36.00	1500.00	8.00
26	36.00	1500.00	7.91
27	36.00	1000.00	8.011
28	12.00	2500.00	5.83
29	48.00	2000.00	7.90
30	48.00	2000.00	7.96

Table S3. Central composite design and response for TPC value in corn tassel extract by mixed enzyme-assisted method

Run	Temprature (°C) (χ_1)	pH (χ_2)	TPC (mg/g)
1	40.00	5.00	10.72
2	47.50	6.00	7.84
3	47.50	5.00	8.04
4	47.50	6.00	7.87
5	40.00	6.00	10.49
6	47.50	6.00	7.90
7	55.00	7.00	6.95
8	55.00	5.00	7.37
9	47.50	6.00	7.83
10	40.00	7.00	10.26
11	55.00	6.00	7.11
12	47.50	7.00	7.77
13	47.50	6.00	7.90

Table S4. Regression coefficients of the fitted second order polynomials representing the relationship between the responses and variables

Factor	Cellulase				Protease				Cellulase-protease mixture			
	Coefficients Estimate	Sum of Squares	F-Value	*P*-value Prob > F	Coefficients Estimate	Sum of Squares	F-Value	*P*-value Prob > F	Coefficients Estimate	Sum of Squares	F-Value	*P*-value Prob > F
Intercept	9.57	37.59	84.84	< 0.0001	7.89	38.63	55.69	< 0.0001	7.87	19.87	1873.48	< 0.0001
χ_1	0.76	8.10	91.39	< 0.0001	1.24	17.00	122.56	< 0.0001	-1.67	16.80	7921.46	< 0.0001
χ_2	0.57	5.70	64.35	< 0.0001	0.066	0.062	0.45	0.5087	-0.19	0.22	103.93	< 0.0001
$\chi_1 \chi_2$	-0.15	0.25	2.84	0.1049	-0.18	0.33	2.35	0.1381	0.010	0.0004	0.19	0.6772
χ_1^2	-1.47	10.59	119.51	< 0.0001	-1.50	9.85	70.99	< 0.0001	0.93	2.37	1117.16	< 0.0001
χ_2^2	-0.56	1.41	15.96	0.0005	0.29	0.32	2.32	0.1408	0.031	0.002	1.27	0.2972
Residual		2.13				3.33				0.015		
Lack of Fit		1.29	2.58	0.0508		1.55	1.73	0.1662		0.011	3.29	0.1399
Pure Error		0.84				1.78				0.004		
Cor Total		39.71				41.96				19.88		
Std. Dev.		0.30				0.37				0.046		
Mean		8.36				7.23				8.31		
C.V. %		3.56				5.15				0.55		
PRESS		3.43				6.94				0.11		
R^2		0.95				0.9206				0.9993		
Adj- R^2		0.94				0.9041				0.9987		
Pred - R^2		0.91				0.8347				0.9943		
Adeq Precision		26.461				19.619				119.220		

χ_1 and χ_2 refer to incubation time and enzyme concentration for cellulase or protease-assisted extraction, and incubation temperature and pH for cellulase - protease mixture-assisted extraction.

Table S5. Antibacterial activity of corn tassels extracts by different extraction methods.

Bacteria	Inhibition zone (mm)					
	Negative control	Positive control	Ethanol	Cellulase	Protease	Enzymatic mixture (1:1)
B. cereus	0	10.5±0.50 ^{a}	6.1±0.52 ^{c}	9.0±0.50 ^{b}	8.7±0.58 ^{b}	10.5±0.50 ^{a}
Staph. Aureus	0	11.8±0.76 ^{a}	5.7±0.56 ^{d}	9.0±0.50 ^{c}	8.8±0.76 ^{c}	10.2±0.44 ^{b}
E.coli	0	23.8±1.25 ^{a}	7.9±0.27 ^{d}	12.2±0.28 ^{b}	10.5±0.50 ^{c}	12.8±0.74 ^{b}
S. typhi	0	15.3±1.04 ^{a}	6.8±0.42 ^{d}	9.5±0.50 ^{bc}	9.0±0.50 ^{c}	10.8±0.60 ^{b}
P. aeruginosa	0	16.2±1.04 ^{a}	7.1±0.38 ^{e}	10.2±0.28 ^{c}	9.3±0.28 ^{d}	11.5±0.50 ^{b}

Values are expressed as mean ± standard error (n=3). Different superscripts within each row are significantly different at $P < 0.05$. Negative control: DMSO, positive control: ceftriaxone.

Table S6. Antifungal activity of corn tassels extracts by different extraction methods.

Fungi	Inhibition zone (mm)					
	Negative control	Positive control	Ethanol	Cellulase	Protease	cellulase-protease mixture (1:1)
A. flavus	0	15.3±1.04 ^{a}	6.1±0.26 ^{d}	8.5±0.50 ^{c}	8.3±0.28 ^{c}	9.0±0.50 ^{b}
A. parasiticus	0	18.2±1.08 ^{a}	6.3±0.37 ^{c}	8.8±0.36 ^{b}	8.5±0.50 ^{b}	8.8±028 ^{b}
A. niger	0	21.2±0.76 ^{a}	6.8±0.31 ^{e}	9.2±0.28 ^{c}	8.3±0.58 ^{d}	10.7±0.76 ^{b}
A. carbonarius	0	15.0±1.50 ^{a}	7.2±0.51 ^{d}	8.5±0.50 ^{c}	8.5±0.86 ^{c}	9.7±0.76 ^{b}
A. ochraceus	0	14.8±0.76 ^{a}	6.4±0.41 ^{d}	8.5±0.50 ^{c}	8.3±0.58 ^{c}	9.8±0.52 ^{b}
A. westerdijikia	0	21.3±0.76 ^{a}	7.1±0.32 ^{e}	10.2±0.76 ^{c}	9.3±0.28 ^{d}	11.0±0.50 ^{b}
F. proliferatum	0	11.7±0.58 ^{a}	6.6±0.48 ^{c}	9.0±0.86 ^{b}	8.8±0.76 ^{b}	9.0±1.00 ^{b}
P. verrucosum	0	20.0±1.00 ^{a}	6.8±0.23 ^{d}	9.8±1.04 ^{b}	8.8±0.76 ^{c}	10.0±0.50 ^{b}

Values are expressed as mean ± standard error (n=3). Different superscripts within each row are significantly different at $P < 0.05$. Negative control: DMSO, positive control: ceftriaxone.

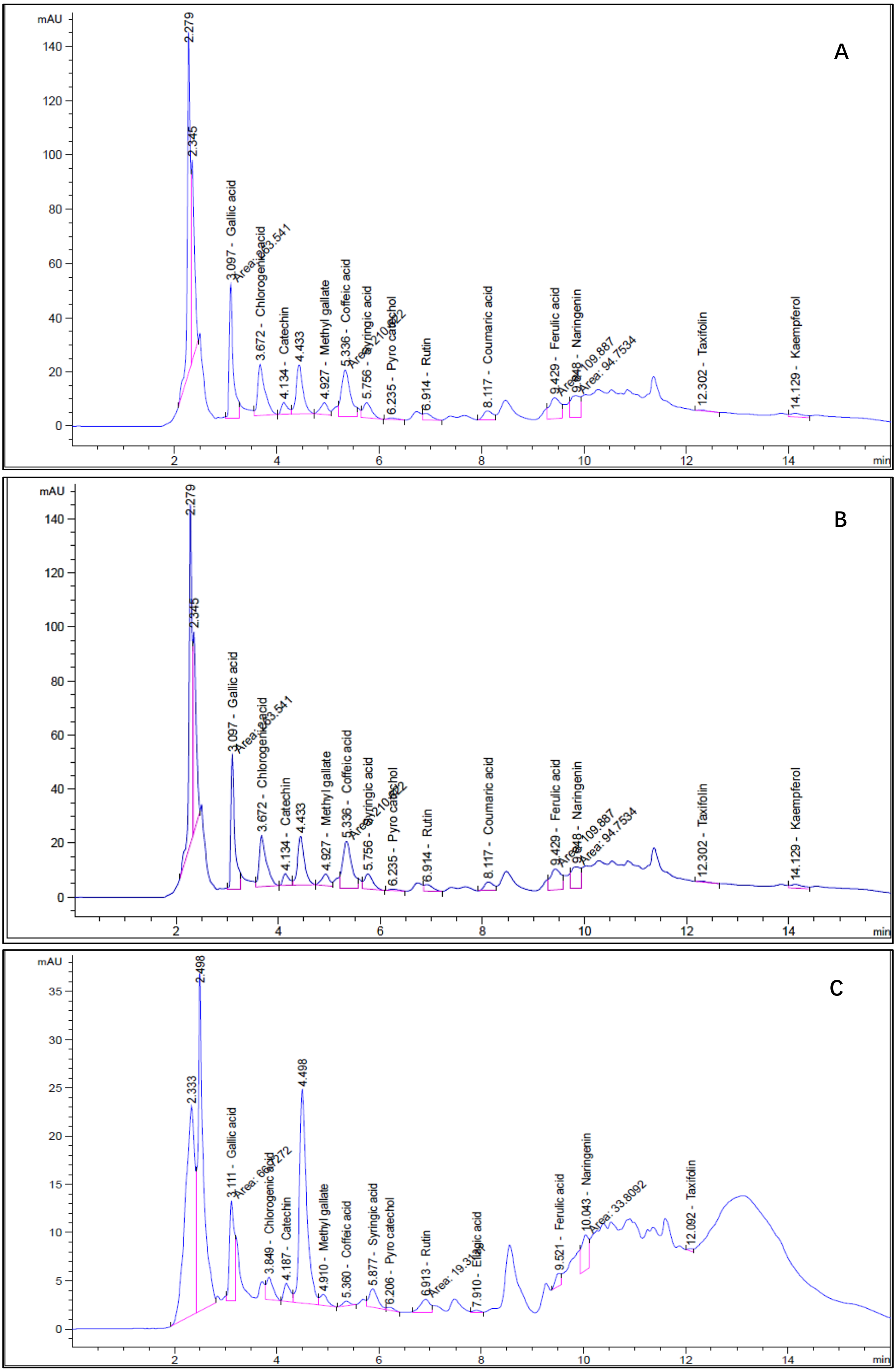

Figure S1. HPLC chromatograms for phenolic compounds in corn tassel extracts by enzyme assisted method. A: Cellulase; B protease; C: cellulase-protease mixture (1:1).
